# Significance of colonization by antibiotic-resistant organisms prior to congenital heart disease surgery in children from low- to middle-income countries sent by non-governmental organizations to Switzerland

**DOI:** 10.1007/s15010-024-02251-8

**Published:** 2024-04-18

**Authors:** Vladimir L. Cousin, Leonce Mwizerwa, Raphael Joye, Noémie Wagner, Tomasz Nalecz, Maya Bouhabib, Tornike Sologashvili, Julie Wacker, Jacques Schrenzel, Maurice Beghetti, Angelo Polito

**Affiliations:** 1 Paediatric Intensive Care Unit, Department of Paediatrics, Gynecology and Obstetrics, Geneva University Hospital, Geneva University of Medicine, Rue Gabrielle-Perret-Gentil 4, 1206 Geneva, Switzerland; 2 Paediatric Cardiology Unit, Department of Paediatrics, Gynecology and Obstetrics, Geneva University Hospital, Geneva, Switzerland; 3 Paediatric Infectiology Department of Paediatrics, Gynecology and Obstetrics, Geneva University Hospital, Geneva, Switzerland; 4 Paediatric Cardiac Surgery Unit, Surgery Department, Geneva University Hospital, Geneva, Switzerland; 5 Bacteriology and Genomic Research Laboratories, Division of Infectious Diseases, Geneva University Hospital, Geneva, Switzerland

**Keywords:** Pediatric intensive care unit, Antibiotic resistance, Low- and middle-income countries, Nosocomial infection, Screening swabs

## Abstract

**Purpose:**

Children with congenital heart disease (CHD) from low- to middle-income countries (LMIC) are suspected to have a high prevalence of antibiotic-resistant microorganisms (ARMOs) carriage, but data are currently lacking. Carriage of ARMOs could impact the post-operative course in pediatric intensive care unit (PICU). The aim of the study was to assess the prevalence of ARMOs carriage in children with CHD from LMIC and its impact on post-operative outcomes.

**Methods:**

This was a retrospective monocentric study from 01/2019 to 12/2022. Included patients were children (0–18 years) from a LMIC admitted after CHD surgery and with AMRO screening performed the week before. Infections and post-operative evolution were compared based on ARMOs carriage status.

**Findings:**

Among 224 surgeries (median age 38.5 months (IQR 22–85.5)), ARMOs carriage was evidenced in 95 cases (42.4%). Main organisms isolated were Extended Spectrum Beta-Lactamase (ESBL) producing *E. coli* (75/224) 33.5%)) and ESBL-*K. pneumoniae* (30/224) 13.4%)). Median mechanical ventilation duration was 1 day (IQR 0–1), PICU stay 3 days (IQR 2–4) and hospital stay 6.5 days (IQR 5–10). A total of 17 infectious episodes occurred in 15 patients, mostly consisting in hospital-acquired pneumonia (HAP) (12/17). Only two infections were caused by a colonizing ARMO. Occurrence of infections and patients’ outcome were similar between ARMO carriers and non-carriers. Higher use of carbapenems (6 (6.3%) vs 1 (0.8%), *p* = 0.04) and a trend to a higher use of vancomycin (14 (13.7%) vs 9 (6.9%), *p* = 0.04) in case of ARMOs carriage. Applying current guidelines, negative swab screening could have led to sparing most of empirical vancomycin therapy (11/12) for HAP based on current guidelines.

**Conclusion:**

Prevalence of AMROs carriage is high in children from LMIC and has a limited impact on patients’ outcome. However, ARMOs carriage leads to higher consumption of antibiotics. Screening may help saving use of broad-spectrum antibiotic in non-carrier patients.

**Supplementary Information:**

The online version contains supplementary material available at 10.1007/s15010-024-02251-8.

## Introduction

Antibiotic-resistant microorganisms (AMROs) colonization rate in patients from low- to middle-income countries (LMIC) has been reported to be high, reaching 50% or more depending on the bacterial species [1]. Among such patients, children from LMIC have similar AMROs colonization rates than adults [1, 2]. The impact of AMROs carriage on outcomes in patients admitted to pediatric intensive care unit (PICU) is not well known, especially for children coming from LMIC. As nosocomial infections prevalence ranges from 10 to 30% in PICU, the risk of inadequate antibiotic therapy is higher in case of unknown AMROs carriage and has been linked to worse clinical outcome in PICU [3–5]. A better knowledge of AMROs carriage in LMIC children is needed to assess the risk and better use of antibiotics, especially in case of empirical antibiotic therapy [6].

Congenital heart disease (CHD) occurs in nearly 1 over 100 children at birth and a significant proportion of children with CHD require complex surgical procedures, which may not be available in LMIC [7]. Non-governmental organizations (NGOs) provide help and access to surgical treatments performed in developed countries [7]. These children often suffer from malnutrition, prolonged cyanosis, and are significantly older than CHD patients from high-income countries. The impact of ARMOs carriage on the post-operative course in PICU in this population of patients is largely unknown.

Geneva Children’s Hospital has an enduring partnership with several NGOs and a long history of providing care for children with CHD from LMIC. Therefore, we sought to assess the prevalence of AMROs carriage prior to cardiac surgery in children with CHD coming from LMIC through NGOs for CHD surgery at our center. We also sought to describe the impact of the AMROs carrier status on the post-operative outcome during their hospital stay.

## Methods

### Study design and population

This retrospective cohort study occurred between January 2019 and December 2022 at the Paediatric Intensive Care Unit (PICU) of Geneva University Hospitals. The study was registered and approved by the local ethic committee (Commission Cantonale d’Ethique de la Recherche de l’Etat de Geneve), project number CCER 2023-00130. This study adheres to national regulations on medical research and complies with the Helsinki Declaration. The ethics committee waived the need for consent as no intervention was performed and screening swabs were an integral component of standard clinical practice.

Primary outcome was the presence of ARMOs, MRSA, or ESBL-E carriage. Secondary outcomes were the total antibiotic consumption and length of hospital stay, evaluated according to the presence of ARMOs, MRSA, or ESBL-E carriage. The use of antibiotics and the impact of screening swabs on the utilization of specific antibiotics (carbapenems and vancomycin) and on the choice of empirical antibiotic therapy in case of hospital-acquired pneumonia (HAP) were assessed. According to our internal policy, admission screening swabs are mandatory in the following cases: patients transferred from another healthcare institution, patient admitted as part of a humanitarian program or patients admitted in a foreign healthcare institution in the previous 12 months.

Study population was composed of all pediatric patients (0–18 years) admitted to the Geneva Children’s Hospital PICU for CHD surgery in the context of HUG–NGOs partnership. Upon admission, patients undergo a thorough evaluation conducted by pediatric cardiologists, pediatric cardio-thoracic surgeons, and cardiac anesthetists to determine the optimal timing for surgery. Due to the critical nature of their conditions, all patients are categorized as either requiring urgent surgery, which occurs within 24 h of admission and pediatric cardiology review, or semi-urgent surgery, performed within 7 days. This classification is essential as the patients typically present in precarious states, necessitating immediate intervention due to advanced congenital heart diseases and limited physiological reserves. Patients with incomplete microbiological (incomplete screening) data or patients who did not undergo surgery for CHD were excluded from the study.

### Data collection

Demographic and clinical variables of interest were collected, including age, weight and body mass index (BMI), country of origin, presence of comorbidities and genetic syndromes, Risk Adjustment for Congenital Heart Surgery (RACHS) score [8], cardiopulmonary by-pass and aortic cross-clamp time, and number of previous surgeries if known. Microbiological data were extracted from patient files including the presence of AMROs and AMROs species if present.

The clinical course in PICU regarding the occurrence of infection, the use of empirical antibiotic therapy, and timing of infection if any were registered.

### Microbiology

AMROs of interest included Methicillin-Resistant *Staphylococcus aureus* (MRSA), Extended Spectrum Beta-lactamase producing *Enterobacteriaceae* (ESBL-E) including *Escherichia coli*, *Klebsiella pneumoniae* and *Enterobacter cloacae*, Vancomycin-Resistant *Enterococcus* sp. (VRE), and carbapenemase-producing Enterobacteriaceae (CPE). In case of CPE, specific carbapenemase type was reported if available.

Routine screening included nasal, axillary, and rectal swabs collected the week before surgery. Swabs (eSwab, Copan, Italy) were routinely collected by trained nurses in pediatric wards or PICU. Swabs were plated on the following agar media: CHROMID® MRSA (BioMérieux, France), CHROMID® ESBL (BioMérieux), CHROMID® OXA-48 (BioMérieux) and CHROMID® VRE (BioMérieux). A matrix-associated laser desorption ionization-time of flight mass spectrometry (MBT Compass 4.1, Bruker Daltonics, Germany) identified bacterial colonies according to published procedures [9–11]. Antibiotic susceptibility testing was assessed through disk diffusion methods according to the EUCAST recommendations [12].

For ESBL-E confirmation, a double-disk synergy test was used. For CPE confirmation, the LAMP Eazyplex Superbug CRE system (AxonLab, UK) was used on selected isolates. The identification of carbapenemase genes included the detection of KPC variants (KPC 2 to 15), NDM variants (NDM1 to NDM7), VIM variants (VIM1 to VIM37), OXA-48-like variants (OXA-48, -62, -204 and -244), and OXA-181-like variants (OXA-181 and -232).

For suspected MRSA colonies growing on the CHROMID® MRSA plate, a qPCR assay targeting the *femA* and *mecA* genes was performed [13]

For suspected VRE colonies growing on CHROMID® VRE, the presence of VRE was confirmed by defining the minimum inhibitory concentration (MIC) for vancomycin and teicoplanin.

### Description of infectious episodes

All cases of possible infections were reviewed, and infectious episodes were retrospectively classified as suspected or proven. Suspected infection was defined as an infectious episode suspected by the attending physicians but not fulfilling the criteria of proven infection. Hospital-acquired pneumonia (HAP) was defined as the presence of radiological signs on two successive chests X-rays showing new or progressive lung infiltrates and at least two clinical/biological criteria: new onset of fever, purulent endotracheal aspirate, leucocytes count < 4 G/L or > 12 G/L, increased minute ventilation, or arterial oxygenation decline [14–16]. In the absence of radiological findings, 3 or more biological/clinical signs had to be present [17]. Significant bacterial isolates were defined as ≥ 10^5^ CFU/ml in quantitative endotracheal specimens or ≥ 10^3^ CFU/ml in distal pulmonary samples. As a significant number of patients were intubated for less than 48h, we selected the term HAP over ventilator associated pneumonia as all children were hospitalized for more than 48 h. Bacteremia was defined as isolation of a non-colonizing microorganism in a blood culture sample [14]. Catheter-related infection was defined as the occurrence of a bacteremia while the catheter was in place or in the 48 h following catheter removal, and culture of the same organism on the catheter (≥ 10^3^ CFU bacteria) or central/peripheral positive blood cultures time-lag > 2h [18]. Urinary tract infection (UTI) was defined as ≥ 10^5^ CFU/ml in urine samples with systemic signs of infection (fever, clinical suggestive symptoms) with a urinary catheter in site for more than 2 days [14]. Surgical-site infection was defined as any bacterial isolation from a sterile liquid or tissue sample from a surgical site [14]. Other sites of infection were defined as isolation of a bacterial pathogen in association with clinical symptoms and systemic signs of infection [14].

### Statistical analysis

Median with interquartile range (IQR) and proportion (%) were used for continuous and dichotomous variables respectively. Univariate associations of independent variables with primary and secondary outcomes were assessed as follows: Wilcoxon rank-sum test for continuous data and chi-square test or Fisher exact test for proportions, depending on the number of events. Logistic regression will be used to calculate odds ratio (OR). Sensitivity, specificity, and predictive values were obtained to assess the performance of screening swabs as a predictor of ESBL-E or MRSA infection. Survival curves were computed using Kaplan–Meier methods and compared using Cox proportional hazard model to calculate hazard ratio (HR) to assess the impact of AMROs, ESBL-E, and MRSA carriage on clinical outcomes, adjusted for time-independent variables, present at admission and not impacted by the surgical and medical management of the patient, such as RACHS score and patient weight. Patient weight was chosen over age, due to the significant presence of underweight children in our cohort. All statistical tests were two-sided with a type 1 error of 0.05. Statistical analyses were done with Stata v14.2 (StataCorps, College Station, TX, USA).

## Results

### Population characteristics and country of origin

**Fig. 1:**
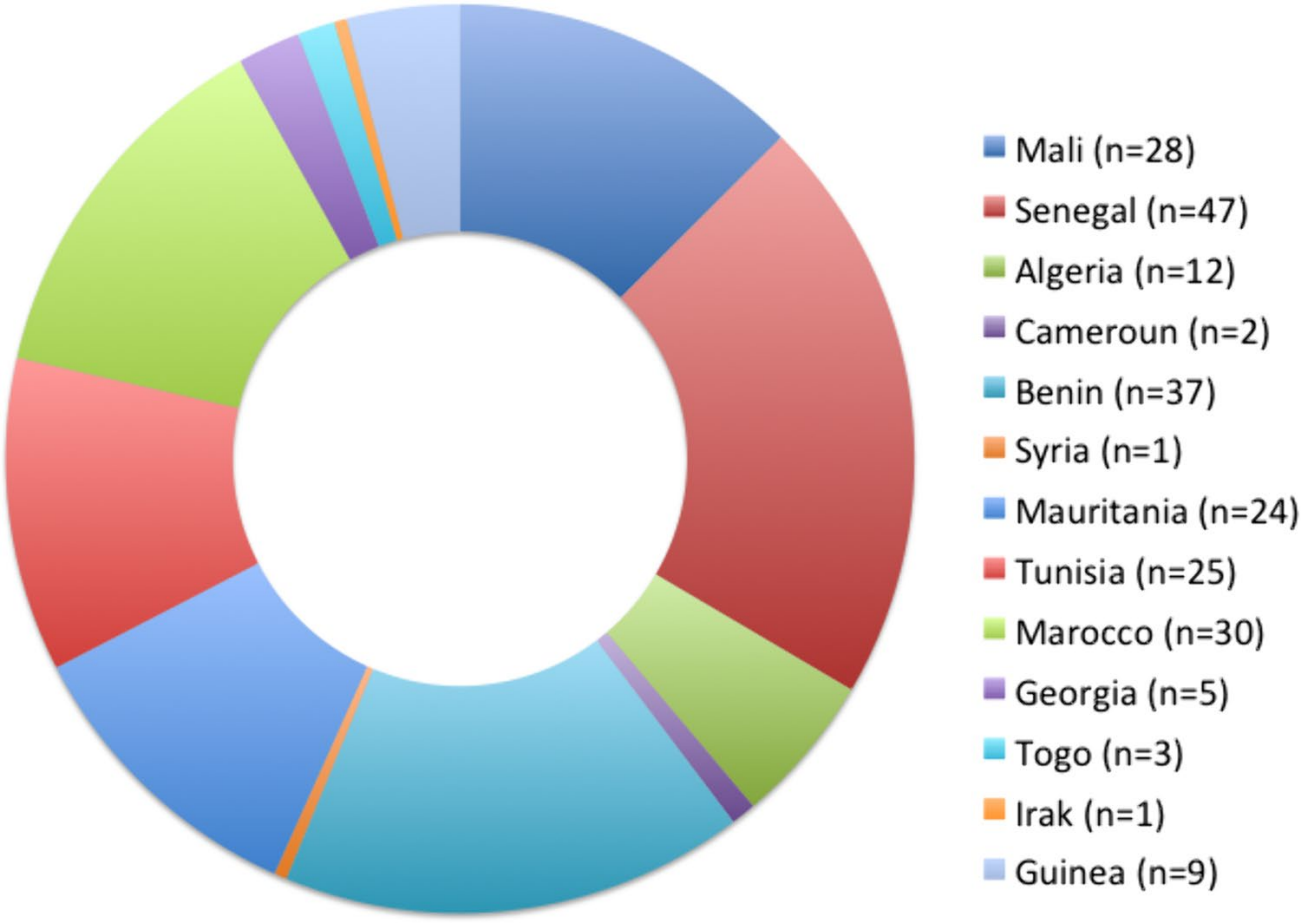
Patients’ countries of origin. Pie chart depicting the country of origin at admission (*N* = 224) of the patients included in the study. Two patients were admitted twice and were from Guinea and Senegal

Over the study period, 226 patients underwent heart surgery and were subsequently admitted in the PICU. Four patients (2%) were excluded from the study due to age-related criteria (2 patients were older than 18 years old at the time of admission) and missed screening swabs (2). This resulted in a final study population of 222 patients, of which a majority did not have a previous CHD surgery 184 (83%), while 29 (13%) had one prior CHD surgery, 6 (2.7%) two, and 3 (1.3%) three surgeries. Countries of origin of the patients are summarized in Fig. 1. The main countries of origin were Senegal (46, 20.7%), Morocco (30, 13.5%), and Tunisia (25, 11.3%).

Baseline characteristics of the study population are presented in Table 1. Median age was 38.5 months (IQR 22–85.5) and 106 (47.3%) had a BMI below the 3rd percentile. Patients with cyanotic CHD accounted for 103 (45.9%) of the cases, and their median oxygen saturation level was 75% (IQR 65–85%). During PICU stay, no deaths were recorded, 2(0.9%) underwent extracorporeal membrane oxygenation (ECMO), and 7 (3.1%) required a delayed sternal closure. Overall, the duration of mechanical ventilation was 1 day (IQR 0–1 day), PICU length-of-stay was three days (IQR 2–4 days), and the total hospital stay averaged 6.5 days (IQR 5–10 days).

**Table 1:** Patient’s characteristics (*n* = 224)

	*N* = 224
Age (months)	38.5 (IQR 22–85.5)
Weight (kg)	11.2 (IQR 8.7–17)
Weight < 3rd percentile	114 (50.9)
BMI (kg/m^2^)	13.4 (IQR 12.4–14.8)
BMI < 3rd percentile	106 (47.3)
Comorbidities	18 (8)
Cyanosis	103 (45.9)
Oxygen saturation (%)	75 (IQR 65–85)
Chronic pulmonary high output	68 (30.3)
RACHS 1	7 (3.1)
RACHS 2	123 (54.9)
RACHS 3	67 (29.9)
RACHS 4	18 (8)
RACHS 6	1 (0.5)
RACHS non-classified	8 (3.6)
Cardiopulmonary bypass (minutes)	80 (IQR 58–115)
Aortic cross-clamp (minutes)	47 (IQR 34–71)
Mechanical ventilation duration (days)	1 (IQR 0–1)
PICU LOS (days)	3 (IQR 2–4)
Hospital LOS (days)	6.5 (IQR 5–10)

Patient characteristics expressed as median (IQR) or number (%)

*BMI* body mass index, *LOS* length of stay, *PICU* pediatric intensive care unit, *RACHS* risk adjustment for congenital heart surgery

### AMRO colonization at ICU admission and impact

**Fig. 2:**
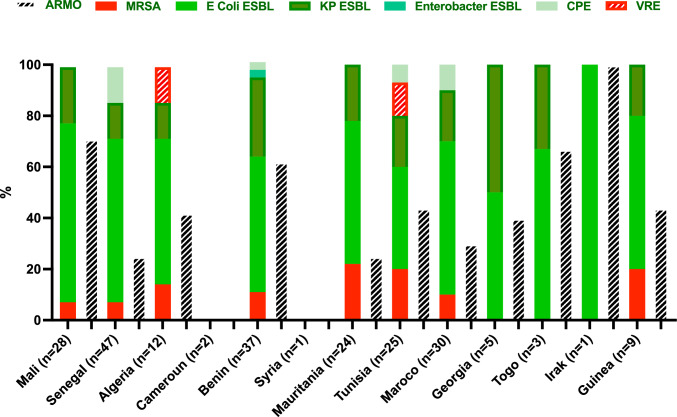
Antibiotic-resistant microorganism detailed by countries. Prevalence of resistant microorganisms by different countries and details of the isolated antibiotic-resistant microorganisms (*AMRO*), including methicillin-resistant *Staphylococcus aureus* (*MRSA*), *Escherichia coli* producing an extended spectrum betalactamase (*E Coli ESBL*), *Klebsiella pneumoniae* producing an extended spectrum betalactamase (*KP ESBL*), *Enterobacter cloacae* producing an extended spectrum betalactamase (*Enterobacter ESBL*), carbapenemase-producing *Enterobacteriaceae* (*CPE*), vancomycin-resistant *Enterococcus* spp. (*VRE*). *N* = for number of patients from this country

At PICU admission, 95 (42.4%) patients were carriers of at least one AMRO and 31 (13.8%) were carriers of multiples AMROs. Main features of isolated AMROs are presented in Table 2. ESBL-E was the most frequent AMRO detected in 85 (37.9%) of the cases, with *Escherichia coli* representing 75 (33.5%) of the cases. Only 5 (2.2%) cases showed carriage of a CPE: 2 in *Escherichia coli* and 3 in *Klebsiella pneumoniae*. The resistance mechanism was a New Delhi metallo-beta-lactamase for 2/5 and an Oxa-181 for 3/5. Prevalence of AMROs carriers varied between patients’ countries of origin, as depicted in Fig. 2. Patients from Cameroun and from Syria did not carry any AMRO but number of patients from these countries was small.

**Table 2:** Details of antibiotic-resistant organisms isolated in screening swabs

Organisms	*N *(%)
AMRO(s)	95 (42.4)
MRSA	15 (6.6)
*Enterobacteriaceae* ESBL	85 (37.9)
*Escherichia coli* ESBL	75 (33.5)
*Klebsiella pneumoniae* ESBL	30 (13.4)
*Enterobacter cloacae* ESBL	1 (0.4)
Vancomycin-resistant *Enterococcus* spp.	3 (1.3)
Carbapenemase producing* Enterobacteriaceae*	5 (2.2)

Number (%) of episode with resistant microbes isolated

*AMRO* antibiotic-resistant organism, *ESBL* extended spectrum beta-lactamase, *MRSA* methicillin-resistant *Staphylococcus aureus*

Table 3 summarizes the main characteristics of AMROs carriers, and MRSA, and ESBL-E carriers. Older (OR 0.99 (95% CI 0.99–0.99), *p* = 0.01) and heavier (OR 0.97 (95% CI 0.95–0.99), *p* = 0.03) patients were significantly less frequently AMROs carriers. Similar characteristics were observed for ESBL-E but not for MRSA carriers. Impact of AMROs, ESBLE-E, and MRSA carriage on clinical outcome is summarized in Table 3. Occurrence of both infection and suspected infection were not statistically different between patients with or without AMROs, MRSA, or ESBL-E carriage. Crude duration of hospital LOS and PICU LOS were significantly different depending on the AMROs and ESBL-E carriers’ status (Table 3). However, AMROs carriers did not have a significant reduction of daily hazard of being discharge from hospital (HR 0.78 (95% CI 0.59–1.01), *p* = 0.06) (supplementary Fig. 1). Similarly, ESBL-E carriers had a non-significant reduction of the daily hazard of being discharged from PICU (HR 0.8 (95% CI 0.6–1.05), *p* = 0.1) or from hospital (HR 0.81 (95% CI 0.61–1.05), *p* = 0.1) (supplementary Fig. 1). Carriage of AMROs and ESBL-E, adjusted for time-independent variables (weight and RACHS category), was not associated with a reduced daily hazard of being discharged from hospital (supplementary Table 1).

**Table 3:** Characteristics and univariate analysis of variable and outcome associated with AMROs, MRSA and ESBL-producing *Enterobacteriaceae* carriage

	No AMRO (*n* = 129)	AMRO (*n* = 95)	*p* value
Age (months)	50 (IQR 27–90)	33 (IQR 18–59)	0.001
Weight (kg)	13 (IQR 9.6–19.6)	10 (IQR 7.7–15)	0.001
BMI (kg/m^2^)	13.6 (IQR 12.6–15)	13 (IQR 12–14.6)	0.04
BMI < 3rd percentile	57 (44.2)	49 (51.6)	0.27
CP bypass (minutes)	80 (IQR 59–117)	80 (IQR 57–109)	0.81
X-clamp (minutes)	48 (IQR 36–74)	46 (IQR 33–66)	0.54
Previous surgery	27 (20.9)	13 (13.7)	0.16
Infection	8 (6.2)	7 (7.4)	0.79
Suspected infection	8 (6.2)	9 (9.5)	0.36
Mechanical ventilation duration (days)	0 (IQR 0–1)	1 (IQR 0–1)	0.17
PICU LOS (days)	3 (IQR 2–4)	3 (IQR 2–5)	0.06
Hospital LOS (days)	5 (IQR 5–9)	7 (IQR 5–12)	0.02

	No MRSA (*n* = 209)	MRSA (*n* = 15)	
Age (months)	38 (IQR 22–84)	40 (IQR 14–95)	0.89
Weight (kg)	11.1 (IQR 8.8–17)	11.6 (IQR 6–21.8)	0.86
BMI (kg/m^2^)	13.5 (IQR 12.5–14.9)	13 (IQR 12–13.6)	0.13
BMI < 3rd percentile	95 (45.5)	11 (73.3)	0.06
CP bypass (minutes)	79 (IQR 57–115)	93 (IQR 74–119)	0.31
X-clamp (minutes)	46 (IQR 34–71)	57 (IQR 30–79)	0.56
Previous surgery	38 (18.2)	2 (13.3)	1
Infection	14 (6.7)	1 (6.7)	1
Suspected infection	15 (7.2)	2 (13.3)	0.32
Mechanical ventilation duration (days)	1 (IQR 0–1)	0 (IQR 0–1)	0.2
PICU LOS (days)	3 (IQR 2–4)	3 (IQR 2–5)	0.72
Hospital LOS (days)	6 (IQR 5–10)	7 (IQR 6–15)	0.42

	No ESBL-E (*n* = 139)	ESBL-E (*n* = 85)	
Age (months)	51 (IQR 27–95)	31 (IQR 18–51)	0.0001
Weight (kg)	13 (IQR 9.6–20.6)	10 (IQR 7.7–13.7)	0.0002
BMI (kg/m^2^)	13.5 (IQR 12.5–15)	13 (IQR 12.1–14.6)	0.12
BMI < 3rd percentile	64 (46)	42 (49.4)	0.62
CP bypass (minutes)	80 (IQR 59–119)	80 (IQR 57–108)	0.69
X-clamp (minutes)	48 (IQR 34–74)	46 (IQR 34–66)	0.56
Previous surgery	28 (20.1)	12 (14.1)	0.25
Infection	9 (6.5)	6 (7)	0.87
Suspected infection	9 (6.5)	8 (9.4)	0.42
Mechanical ventilation duration (days)	0 (IQR 0–1)	1 (IQR 0–1)	0.07
PICU LOS (days)	3 (IQR 2–4)	3 (IQR 2–5)	0.03
Hospital LOS (days)	6 (IQR 4–9)	7 (IQR 5–12)	0.02

Variables are expressed as median (IQR) or number (%)

*AMRO* antibiotic-resistant organism, *CP* bypass cardiopulmonary bypass, *HR* hazard ratio, *OR* odds ratio, *PICU* pediatric intensive care unit, *RACHS* risk adjustment for congenital heart surgery, *X-clamp* aortic clamp

### Infectious event and antibiotic use during PICU

During their stay in PICU, 13 patients had one infectious episode and 2 patients had two episodes. Infections were mostly HAP in 12/17 (70.5%), surgical-site infection in 1 (5.8%), endocarditis in 2 (11.6%), CLABSI in 1 (5.8%), and one sinusitis (5.8%). Timing of infection was 2 days (IQR 0–2) after PICU admission. Suspected infection occurred in 17 patients, suspected infectious sources being sepsis in 7/17 (41.2%) and HAP in 10/17 (58.8%). Neither AMRO nor ESBL-E or MRSA carriage was associated with an increased risk of infection.

Two infectious episodes were caused by an ESBL-E and MRSA respectively. The performance of ESBL-E screening and MRSA screening assessed through a ROC curve construction was 0.97 (95% CI 0.94–0.98) for MRSA and 0.81 (95% CI 0.76–0.86) for ESBL. For both MRSA and ESBLE-E, screening swabs showed high sensitivity and negative predictive value (supplementary Table 2).

Regarding antibiotic therapy, vancomycin was used in 22 (9.8%) of patients, with a median duration of therapy of 2 days (IQR 2–4) and a consumption of 85.9 days of vancomycin/1000 PICU days (Table 4). Carbapenems were used in 7 (3.1%) patients, for a median duration of 4 days (IQR 3–10) and a consumption of 42.9 days of carbapenems/1000 PICU days. AMROs carriers had a higher consumption of carbapenems (OR 8.6 (95% CI 1.02–72.9, *p* = 0.04) but not of vancomycin (OR 2.3 (95% CI 0.95–5.5), *p* = 0.06). Carriage of MRSA was associated with a higher risk of vancomycin consumption (OR 5.3 (95% CI 1.6–17.2), *p* = 0.005) while ESBL-E carriers were at higher risk of carbapenems consumption (10.4 (95% CI 1.2–88.6), *p* = 0.03) (Table 4).

**Table 4:** Antibiotic consumption based on AMRO, ESBL *Enterobacteriaceae* and MRSA carriage status

	No AMRO (*n* = 129)	AMRO (*n* = 95)	*p* value	OR (95% CI)
At least 1 vancomycin day	9 (6.9)	14 (13.7)	0.06	2.3 (0.95–5.5) 0.06
Duration vancomycin (days)	2 (IQR 2–3)	2 (IQR 2–5)	0.67	
Duration of vancomycin (/1000 PICU days)	58.5	116.9	0.05	
At least 1 carbapenem day	1 (0.8)	6 (6.3)	0.04	8.6 (1.02–72.9) 0.04
Duration carbapenem (days)	3 (IQR 3–3)	5.5 (IQR 3–10)	0.29	
Duration of carbapenem (/1000 PICU days)	6	84.8	0.01	

	No MRSA (*n* = 209)	MRSA (*n* = 15)		
At least 1 vancomycin day	18 (8.6)	5 (26.7)	0.01	5.3 (1.6–17.2) 0.005
Duration vancomycin (days)	2 (IQR 2–4)	2 (IQR 2–2)	0.56	
Duration of vancomycin (/1000 PICU days)	74.4	263.2	0.001	

	No ESBL (*n* = 139)	ESBL (*n* = 85)		
At least 1 carbapenem day	1 (0.7)	6 (7)	0.01	10.4 (1.2–88.6) 0.03
Duration carbapenem (days)	3 (IQR 3–3)	5.5 (3–10)	0.29	
Duration of carbapenem (/1000 PICU Days)	5.6	92.5	0.008	

**Fig. 3:**
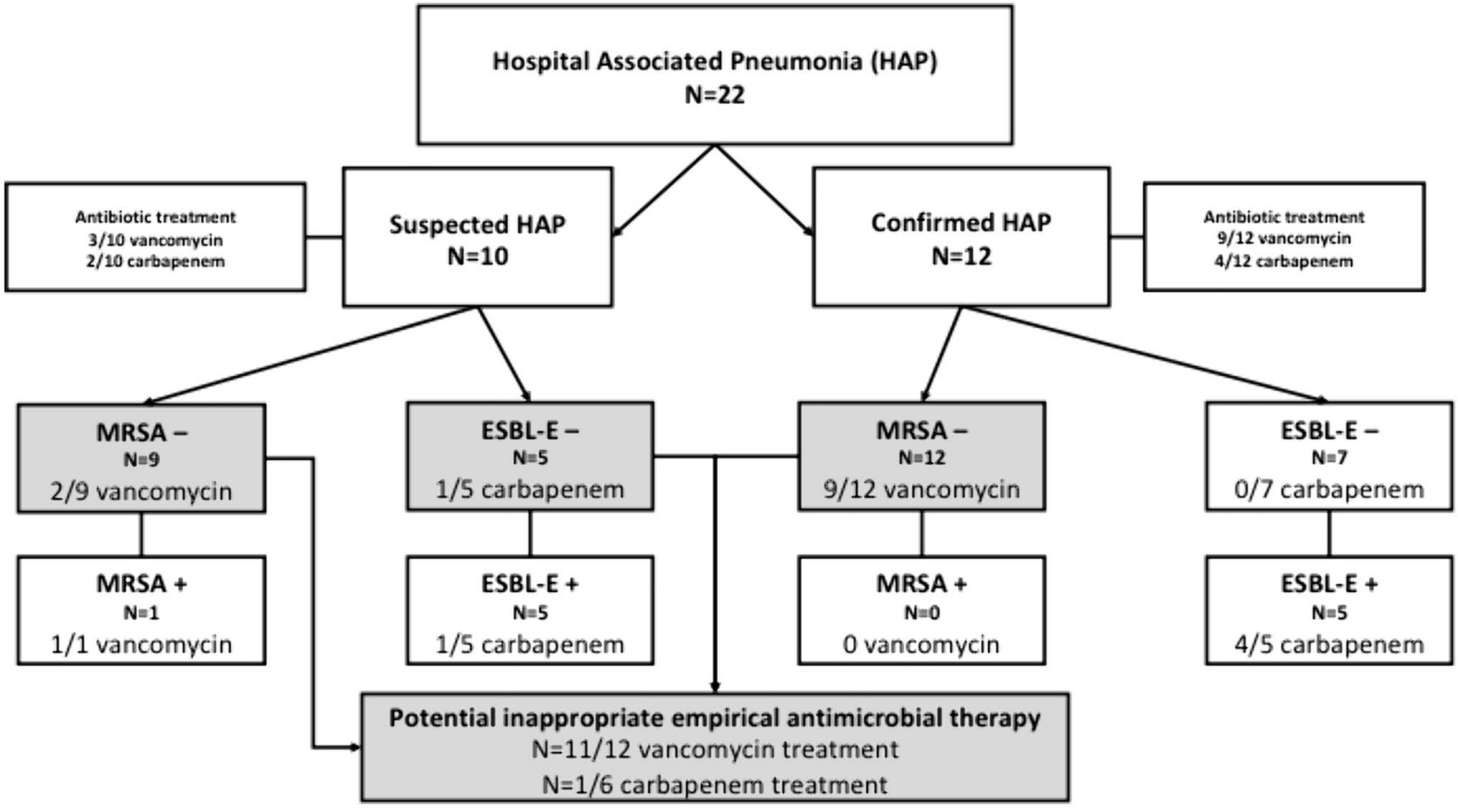
Potential impact of AMROs screening on empirical antibiotic therapy. Empirical antibiotic therapy in patients with potential HAP after the cardiac surgery and suggestion to limit the use of vancomycin/carbapenem in the setting of empirical antibiotic therapy based on best practice recommendation on HAP/VAP [7, 29, 30]. In each box, presence ( +) or absence (−) of the colonizing AMRO, either MRSA or ESBL-E, is specified. Number (*N* =) of patients and the number of patients treated with vancomycin or carbapenem (a/N vancomycin treatment or a/N carbapenem treatment). In gray, potential vancomycin/ carbapenem overuse for a HAP treatment. *AMRO* antibiotic-resistant organism, *ESBL* extended spectrum beta-lactamase, *HAP* hospital-acquired pneumonia, *MRSA* methicillin-resistant *Staphylococcus aureus*

Clinical impact of strict application of antibiotic stewardship best practice guidelines on empirical antibiotic therapy in suspicion of HAP was assessed as it was by far the most frequent cause of antibiotics’ initiation. Figure 3 compares the empirical antibiotic prescription of vancomycin and carbapenem with the suggested use of those antibiotics based on the ESBL-E or MRSA carriage status. Most of vancomycin therapy (11/12, 92%) may have been spared in cases of suspected or confirmed HAP. For carbapenem prescription, one utilization could have been avoided (1/6, 16%).

## Discussion

Our findings highlight the concerning prevalence of antibiotic-resistant bacteria among children from LMIC, with ESBL-producing *Escherichia coli* as the predominant strain. Interestingly, AMROs were rarely responsible for infections, and AMROs carriage had minimal impact on patient outcomes. Importantly, screening swab may improve the empirical antibiotic therapy, especially for HAP, and help limit the use of carbapenems and vancomycin.

The significant difference in the prevalence of AMROs carriage between high-income countries and LMIC is becoming an increasingly important global healthcare issue [19]. Yet, we only have limited information about the extent of antibiotic resistance in LMIC as data are lacking. The increased prevalence of antibiotic resistance in these countries is thought to be due to the misuse of antibiotics, crowded conditions, and inadequate measures to prevent and control infections [2]. In children with CHD, the likelihood of carrying AMROs can increase due to various factors, such as frequent exposure to healthcare settings and repeated use of antibiotics for respiratory issues. We provide one of the first detailed account of AMROs carriage among children with CHD in various LMICs [2, 20]. We assessed the significant occurrence of AMROs, specifically ESBL-producing *E. coli* and *K. pneumoniae*, within this group of patients. MRSA represented only a small proportion of AMROs [2, 19]. The growing problem of antibiotic resistance in Gram-negative bacteria poses a significant challenge for future medical treatment of infections, particularly in patients from LMICs [20]. In LMIC, the emergence of resistant microorganisms threatens the effectiveness of currently recommended empirical antibiotic therapies for several conditions including neonatal sepsis [6].

Although ARMOs are quite common, they were seldom found to be the main infectious agents in our group of patients. Even though the occurrence of AMROs infections in the PICU is increasing, it only represents a small portion of infectious cases in wealthy countries [21]. These infections typically occur after prolonged stays in intensive care and multiple rounds of antibiotic therapies, which was not often the case in our study population [22–24]. A possible reason for the lower-than-expected occurrence of infections after surgery in our cohort of children with CHD might be the limited duration of mechanical ventilation in the post-operative period [5].

Admission screening for AMROs has been shown to potentially improve the empirical antibiotic therapy, thanks to an excellent negative predictive value [22, 25, 26]. Even if the number of patients with ESBL-E or MRSA infectious episodes was limited in our population, both ESBL-E and MRSA colonization had high sensitivity and negative predictive value. Of note, evidence of ESBL-E or MRSA carriage was associated with more use of broad-spectrum antibiotics, which was consistent with previous reports [22, 27, 28]. In areas with low incidence of antibiotic-resistant infections, the abandonment of routine testing of admitted patients has allowed to reduce the use of broad-spectrum antibiotics, with similar outcome [29]. However, regular screening is still recommended in populations where AMROs are common. This screening can assist in tailoring antibiotic therapies more effectively, particularly when AMROs are not present. Additionally, screening can help prevent the spread of AMROs [4, 30].

Both in PICU and cardiac intensive care unit, HAP is one the most frequent nosocomial infection [5, 21]. In our cohort, HAP was the main cause of antibiotic therapy initiation. We explored the potential impact of rigorously adherence to current guidelines for negative screening swabs on the empirical administration of vancomycin and carbapenems for probable or established HAP. Building upon prior studies, the high negative predictive value of the test in our cohort indicates its potential on sparing broad-spectrum antibiotic therapies for 3rd generation resistant Enterobacteriaceae, ESBL-E and for MRSA. [23, 26, 31]. A decision tree analysis suggests that the use of many wide-ranging antibiotic therapies, particularly vancomycin, might be avoided [4, 16, 17, 32]. Moreover, in our population MRSA and ESBL-E were only isolated in carriers. To curtail the unnecessary use of broad-spectrum antibiotics in HAP and minimize their impact on the PICU ecology and AMRO spread, our findings advocate for the implementation of a negative screening swab to inform the judicious administration of vancomycin or carbapenems during the initiation of empirical antibiotic therapy for HAP, alongside adjustments based on the local bacterial ecology.

The strengths of this study lay in its comprehensive screening of a large, previously unreported cohort of children with CHD in LMIC, yielding valuable insights into AMRO carriage among LMIC pediatric patients. However, the study's retrospective and monocentric design may limit the generalizability of its findings, particularly to PICUs with diverse patient populations and local ecologies. Additionally, the reliance on multisite swabbing, which may not have perfect sensitivity, could have impacted the accuracy of carriage assessment. Finally, the relatively low number of infections caused by multidrug-resistant organisms may have hindered our ability to fully elucidate the association between carriage and patient outcomes.

## Conclusion

The carriage of AMROs, particularly ESBL-E, is common among children with CHD from LMICs. In the present study, AMRO carriage does not significantly affect patients' outcomes, but leads to higher consumption of antibiotics. Screening swab could have a role to limit such consumption, but evidence of clinical benefit of screening swab in this specific population warrants further studies.

## Supplementary Information

Below is the link to the electronic supplementary material.

Supplementary file1 (DOCX 298 KB)

## Data Availability

Data may be available upon reasonable requests to the contact author and the senior author of this manuscript.

## Abbreviations

AMRO: Antibiotic-resistant microorganisms
BMI: Body mass index
CPE: Carbapenemase producing *Enterobacteriaceae*
CHD: Congenital heart disease
ESBL-E: Extended spectrum beta-lactamase producing *Enterobacteriaceae*
Enterobacter ESBL: *Enterobacter cloacae* producing an extended spectrum betalactamase
E Coli ESBL: *Escherichia coli* producing an extended spectrum betalactamase
HAP: Hospital-acquired pneumonia
IQR: Interquartile range
KP ESBL: *Klebsiella pneumoniae* producing an extended spectrum betalactamase
LMIC: Low- to middle-income countries
MIC: Minimum inhibitory concentration
MRSA: Methicillin-resistant *Staphylococcus aureus*
PICU: Pediatric intensive care unit
RACHS: Risk adjustment for congenital heart surgery
VRE: Vancomycin-resistant *Enterococcus* spp.
