# Supplementary material for: Significance of colonization by antibiotic-resistant organisms prior to congenital heart disease surgery in children from low- to middle-income countries sent by non-governmental organizations to Switzerland
Source: Infection. 2024 Apr 18;52(5):1889–99. doi: 10.1007/s15010-024-02251-8 (PMC11499515; doi:10.1007/s15010-024-02251-8)
Supplement: Supplementary file 1 — Supplementary file1 (DOCX 298 KB) [file 15010_2024_2251_MOESM1_ESM.docx]

**Supplementary figure 1**

Probability of being discharge from PICU or hospital, depending on the ARMOs carrier or ESBL-E carrier status.

Panel A PICU LOS depending on the AMROs carrier status

Panel B Hospital LOS depending on the AMROs carrier status

Panel C PICU LOS depending on the ESBL-E carrier status

Panel D Hospital LOS depending on the ESBL-E carrier status

AMRO antibiotic resistant organism; ESBL extended spectrum beta-lactamase; LOS length of stay; MRSA methicillin resistant *Staphylococcus aureus*

**Supplementary table 1**

**Multivariate model addressing the hazard of being discharge from PICU of hospital, based on the presence of AMRO or ESBL-E carriage**

|  | Hospital discharge |
| --- | --- |
| AMRO carriage | 0.9 (0.68-1.18) 0.45 |
| Weight 8.76-11.2 kgs | 2.03 (1.38-2.9) <0.001 |
| Weight 11.3-17 kgs | 2.43 (1.63-3.6) <0.001 |
| Weight >17 kgs | 1.98 (1.34-2.9) 0.001 |
| RACHS >=3 | 0.66 (0.50-0.88) 0.004 |
| shoefield 0.18 |  |
|  |  |
|  | Hospital discharge |
| ESBL-E carriage | 0.99 (0.74-1.32) 0.96 |
| Weight 8.76-11.2 kgs | 2.07 (1.40-3.04) <0.001 |
| Weight 11.3-17 kgs | 2.49 (1.67-3.7) <0.001 |
| Weight >17 kgs | 2.02 (1.36-3.01) 0.001 |
| RACHS >=3 | 0.67 (0.51-0.88) 0.004 |
| shoefield 0.16 |  |
|  |  |
|  | PICU discharge |
| AMRO carriage | 0.94 (0.71-1.24) 0.68 |
| Weight 8.76-11.2 kgs | 1.6 (1.13-2.47) 0.01 |
| Weight 11.3-17 kgs | 1.84 (1.24-2.73) 0.002 |
| Weight >17 kgs | 2.05 (1.37-3.07) <0.001 |
| RACHS >=3 | 0.71 (0.54-0.94) 0.01 |
| shoefield 0.77 |  |
|  |  |
|  | PICU discharge |
| ESBL-E carriage | 0.94 (0.71-1.25) 0.68 |
| Weight 8.76-11.2vkgs | 1.67 (1.13-2.47) 0.009 |
| Weight 11.3-17 kgs | 1.84 (1.24-2.73) 0.002 |
| Weight >17 kgs | 2.04 (1.36-3.08) 0.001 |
| RACHS >=3 | 0.71 (0.54-0.94) 0.01 |
| shoefield 0.81 |  |

Multivariate analysis hazard model to assess impact of multiple drug resistant organisms (MDROs) and extended spectrum betalactamase producing *Enterobacteriaceae* (ESBL-E) on paediatric intensive care unit (PICU) and hospital length of stay. Adequacy of the model was assessed with Shoefield residuals.

**Supplementary table 2**

**Performance characteristics of ESBL-E and MRSA admission screening swab as a predictor of ESBL-E or MRSA infection.**

|  | Sensitivity  % (95% CI) | Specificity  % (95% CI) | Positive Predictive Value  % (95% CI) | Negative Predictive Value  % (95% CI) |
| --- | --- | --- | --- | --- |
| ESBL-E infection | 100 (5.1-100) | 62.3 (55.8-68.4) | 1.1 (0.06-6.3) | 100 (97.3-100) |
|  |  |  |  |  |
| MRSA infection | 100 (5.1-100) | 93.7 (89.7-96.2) | 6.7 (0.34-29.8) | 100 (98.2-100) |

Performance of screening swab to predict infection by a MRSA or a ESBL-E. Extended spectrum batalactamase producing *Enterobacteriaceae* (ESBL-E); methicillin resistant *Staphylococcus aureus* (MRSA).
